# Death anxiety, resilience, and family cohesion in parents of children and adolescents in the end stages of life

**DOI:** 10.3389/fpsyg.2023.1057003

**Published:** 2023-02-10

**Authors:** Fateme Mohammadi, Seyedeh Zahra Masoumi, Khodayar Oshvandi, Mostafa Bijani, Leila Nikrouz

**Affiliations:** ^1^Chronic Diseases (Home Care) Research Center and Autism Spectrum Disorders Research Center, Department of Nursing, Hamedan University of Medical Sciences, Hamedan, Iran; ^2^Department of Midwifery, School of Nursing and Midwifery, Mother and Child Care Research Center, Hamadan University of Medical Sciences, Hamedan, Iran; ^3^Department of Medical-Surgical Nursing, School of Nursing and Midwifery, Mother and Child Care Research Center, Hamedan University of Medical Sciences, Hamedan, Iran; ^4^Department of Medical Surgical Nursing, School of Nursing, Fasa University of Medical Sciences, Fasa, Iran

**Keywords:** death anxiety, family adaptability, cohesion, resilience, parents, children, end stage

## Abstract

**Purpose:**

Adolescents in the end stages of life impose a lot of psychological stress on other family members, which may affect their resilience and quality of life. So, the aim of the present study was to investigate death anxiety, family adaptability and cohesion, and resilience in the parents of children and adolescents who were at the end stages of life.

**Methods:**

This is a cross-sectional study. Two hundred and ten parents were selected by convenience sampling and completed the questionnaires such as demographics survey, the death anxiety scale, Connor-Davidson resilience scale, family adaptability, and cohesion scale. Data were analyzed by descriptive statistics (frequency, percentage, mean, and standard deviation), independent *t*-test, ANOVA, and multiple linear regressions. The significance level was set at *p* < 0.05.

**Result:**

The findings showed that death anxiety in parents of children and adolescents in the end stages of life has a significant inverse correlation with family adaptability and cohesion (*p* < 0.001, *r* = −0.92) and resilience (*p* < 0.001, *r* = −0.90). The variables of family adaptability and cohesion, resilience, number of children, the children’s illness duration, and marital status can predict 61.34% of the death anxiety variance in these parents.

**Conclusion:**

The parents of children and adolescents in end stages of life reported high death anxiety and moderate family adaptability and cohesion, but low resilience. Accordingly, pediatric nurses and healthcare policymakers should develop comprehensive support plans for these parents to facilitate their adaptation and increase their family adaptability and cohesion.

## 1. Introduction

As a very sensitive period of one’s life, adolescence is characterized by various physical, psychological, and social changes. During this period, adolescents go through a variety of cognitive, neurobiological, and social crises (Mohammadi et al., 2016). However, the emergence of a disease especially, chronic diseases can make it increasingly difficult for adolescents to pass through this developmental stage (Holakouie-Naieni et al., 2016) and adversely affects their natural development and quality of life of these teenagers and their families (Langton and Berger, 2011). A review of literature shows that the occurrence and prevalence of chronic diseases among adolescents is increasing in many developing countries, so that one-third of children and adolescents suffer from one or more chronic diseases (Pinquart, 2020). Also, it was predicted that, in 2020, chronic diseases would be the main cause of death in this age group (Reif et al., 2022). Among these, the most common chronic diseases during adolescence are respiratory, cardiovascular, kidney, language-speech-auditory disorders, cancer, and diabetes (Lerch and Thrane, 2019). On the other hand, chronic heart, respiratory, kidney, and cancer diseases are the most important chronic diseases of teenagers, which cause teenagers and their families to endure years of pain and suffering, and eventually cause the death of teenagers in many cases (Wijlaars et al., 2016).

Even though advances in medical sciences and technology have increased the survival rate of adolescents with chronic diseases. So that the most important causes of death of teenagers in Iran are head trauma, heart disease, poisoning, suffocation, and accidents. But chronic diseases especially cancer, cardiovascular diseases, neuromuscular diseases still account for many deaths among adolescents (Holakouie-Naieni et al., 2016). Death is a terrifying prospect for adolescents and parents: the thought of death exposes them to many fears, including fear of the hereafter, leaving their adolescents and parents, and being left alone (Reif et al., 2022). To confront their fears and concerns with regard to death, adolescents and parents ask their parents and care providers for information or even rely on fantasy. Thus, there is an urgent need for immediate interventions on the part of care providers to address death among adolescents, e.g., efforts to prepare adolescents and their parents for death, raising their awareness of peaceful death, reducing their death anxiety, and promoting cohesion in these families (Lerch and Thrane, 2019; Reif et al., 2022).

The parents of children in the end stages of life experience extreme fear and anxiety about the impending death of their offspring. As a multi-dimensional concept, death anxiety is defined as fear of one’s own death or the death of a loved one (Wijlaars et al., 2016). Anticipation of the death of their children is a painful experience for parents and leads to depression, fear, and chaos in the family, with an adverse effect on the parents’ patience and resilience (Ahmadi Farsani et al., 2020). Resilience is a key variable which has a great impact on the mental health of children who are faced with death anxiety and cohesion in their families (Phillips et al., 2020). Although there are contradictory views on concept of resilience, resilience is more defined as an individual’s ability to maintain his/her bio-psychological balance in dangerous situations (Phillips et al., 2020); it is the ability to return to a balanced state or a higher state which results in successful coping (Javalkar et al., 2017). Resilience is a personality trait which enables an individual to live through hard times and stay strong in the face of difficulties (Javalkar et al., 2017; Phillips et al., 2020). Thus, resilience can help reduce stress and feelings of incompetence during hard times, which improves life satisfaction and mental health (Cafferkya et al., 2018). Research shows that, in stressful situations, individuals with greater resilience enjoy better mental health than individuals with lower levels of resilience (Garcia-Dia et al., 2013; Scoloveno, 2016). Thus, the ultimate goal of healthcare for adolescent patients and their parents should be to improve their adaptation, resilience, and quality of life (Windle, 2011). In recent years, several studies have addressed adaptation, resilience, and quality of life in the parents of children with chronic diseases or after the death of their children and adolescents (Wikman et al., 2018; Koca et al., 2019; Cao et al., 2020; Dadfar et al., 2021), especially children with cancer (Toledano-Toledano et al., 2019; Vegsund et al., 2019). But, researchers do not find studies that investigate the relationship between death anxiety and resilience and family cohesion in the parents of children and adolescents who are at the end of life. Accordingly, the present study was conducted to investigate the relationship between death anxiety and resilience and family cohesion in the parents of children and adolescents who are at the end of life. The hypotheses of this study included the following; “parents who have more death anxiety have less resilience” and “parents who have more death anxiety have less family cohesion.”

## 2. Materials and methods

### 2.1. Study design and setting

The present study is a cross-sectional work of research conducted from March to May 2022. This research was reported based on the strengthening the reporting of observational studies in epidemiology (STROBE) statement, that is guidelines for reporting observational studies.

### 2.2. Participants and sampling

In this study, the sample size has been estimated based on the study of Ahmadi Farsani et al. (2020) with *β* = 80% and *α* = 0.05 and taking into account the 20% drop about 250 samples. So, the participants were 250 parents who had children and adolescents in the end stages of life base on medical record in one of the five university hospitals in the south and west of Iran that selected by convenience sampling. The inclusion criteria were being willing to participate in the study and having a child in the end-of-life stage based on medical record. The participants who failed to respond to more than half of the items on the questionnaires or did not return the questionnaires were excluded. The participants were asked to complete a demographics survey, a death anxiety questionnaire, a family cohesion questionnaire, and a resilience questionnaire. All the questionnaires were to be completed online. So, researchers approached 250 parents of these children but, 40 parents did not complete the questionnaires due to the lack of suitable psychological conditions, excessive fatigue, or the death of their child. Thus the final sample consisted of 210 of the participants completed and returned the questionnaires *via* email or a social network (a response rate of 0.84).

### 2.3. Measurements

#### 2.3.1. The demographics survey

The survey addressed the respondents’ age, gender, marital status, their children’s disease type, their children’s illness duration, number of children, financial status, and education.

#### 2.3.2. The death anxiety scale

This scale was designed by Templer in 1970 that consists of 15 true/false items (Tomás-Sábado and Gómez-Benito, 2002). In nine items, “true” earns a score of 1, while in six items, “false” earns a score of 1. The score range is between 1 and 15, with higher scores indicating greater death anxiety. A respondent’s score can be classified into one of the three categories of mild anxiety (0–6), moderate anxiety (Wijlaars et al., 2016; Ahmadi Farsani et al., 2020; Phillips et al., 2020), and severe anxiety (Windle, 2011; Garcia-Dia et al., 2013; Scoloveno, 2016; Javalkar et al., 2017; Cafferkya et al., 2018; Wikman et al., 2018). Sharif Nia et al. (2016) reported this scale has good content and construct validity. Also, Cronbach’s alpha of 0.88 shows that it is a reliable instrument (Sharif Nia et al., 2016).

#### 2.3.3. Connor–Davidson resilience scale

This scale developed by Connor and Davidson (2003), this scale measures an individual’s ability to cope with stressful and threatening situations. The scale consists of 25 items in five domains: personal competence and tenacity (eight items), tolerance of negative affect and strengthening effects of stress (seven items), positive acceptance of change (five items), self-control (three items), and spiritual influences (two items). The items are scored on a 5-point Likert scale: not true at all (0) to almost always true (Pinquart, 2020). The score range is between 0 and 100, with higher scores indicating greater resilience (Connor and Davidson, 2003). Fayand et al. (2019) reported the face and content validity and reliability of the scale to be satisfactory. Reliability of the Cronbach’s alpha of the scale was 0.82 (Fayand et al., 2019).

#### 2.3.4. Family adaptability and cohesion scale

The Family Adaptability and Cohesion Scale was developed by Olson (2011). This scale is a self-assessment tool which measures family performance in two areas: family adaptability and family cohesion. Family cohesion refers to the degree of emotional closeness between the members of a family. Adaptability is defined as stability in the structure and performance of a family when conditions in the family are changing. The scale consists of 20 items: 10 items concern family cohesion and 10 items concern family adaptability. The items are scored on a 5-point Likert scale, ranging from never (Mohammadi et al., 2016) to always (Reif et al., 2022). The face and content validity of the scale has been tested and verified. The reliability of the scale has been found to equal a Cronbach’s alpha of 0.89 (Cong et al., 2022). In this study, the face, content validity and reliability of this scale were evaluated. Reliability of the Cronbach’s alpha of the scale was 0.89.

### 2.4. Ethical considerations

All participants signed the informed consent to participate in the study. The present study was conducted in accordance with the principles of the revised Declaration of Helsinki, a statement of ethical principles which directs the physicians and other participants in medical research involving human subjects. The participants were assured of their anonymity and confidentiality of their information Moreover, the study was approved by the local Ethics Committee of Fasa University of Medical Sciences (Ethical code: IR.FUMS.REC 1401.029).

### 2.5. Statistical methods

The collected data were analyzed using descriptive statistics (frequency, percentage, mean, and standard deviation) in SPSS v. 22. The researchers used the Chi-square test and independent t-test to study the relationship between death anxiety and resilience, family cohesion, and demographic variables in the parents of children and adolescents in the end-of-life stage. Level of significance was set at *p* < 0.05. Next, the variables of demographics, resilience, and family cohesion which were found to correlate with death anxiety (*p* < 0.25) were entered into multivariate linear regression with the backward technique (Mohammadi et al., 2021). Before multivariate linear regression, the researchers tested the assumptions of normality of data, homogeneity of variance, and independence of residuals.

## 3. Results

### 3.1. Demographic information

Out of the 210 parents who participated in the study, 110 (52.39%) were mothers The participants were aged between 27 and 58 years, with the mean being 38.76 ± 3.57 years, and the majority of them were married 198 (94.28). The majority of the children in the present study had cancer 47 (35.24%), Also, about 79 (37.62) of them had been sick for about 12–14 years. Almost half of the participants (46%) of the parents of your study had passed 4–7 years since the diagnosis of their child’s disease. One hundred and twenty-one (57.6%) of the children were boys and 89 (42.39%) were girls. The mean age of the children was 12.84 ± 1.39 years.

The results showed that there was a statistically significant relationship between the parents’ death anxiety for their children and their marital status, number of children, and illness duration of their children (Table 1).

**Table 1:** The participants’ demographic characteristics and death anxiety.

Demographic variables		Number (%)	Death anxiety Means ± SD	*p*-value
Mother’s age (Year)	27–36	74 (35.23)	13.14 ± 1.23	0.901**
	37–46	102 (48.57)	13.43 ± 1.21	
	47–58	34 (16.20)	13.57 ± 1.28	
Mother’s education	Illiterate	10 (4.76)	13.28 ± 1.14	0.892**
	Primary	40 (19.04)	13.11 ± 1.21	
	Diploma	107 (50.96)	12.95 ± 1.05	
	Bachelor	25 (11.90)	12.41 ± 1.24	
	Master’s degree and higher	28 (13.34)	13.13 ± 1.57	
Mother’s job	Self-employed	73 (34.76)	12.78 ± 1.34	0.881**
	Employee	40 (19.04)	13.98 ± 1.68	
	Housewife	97 (46.20)	13.58 ± 1.14	
Father’s age (Year)	27–36	58 (27.61)	12.64 ± 1.39	0.892**
	37–46	73 (34.76)	12.08 ± 1.52	
	47–58	55 (26.19)	12.94 ± 1.13	
Father’s education	Illiterate	13 (13.34)	11.43 ± 1.21	0.791**
	Primary	45 (21.43)	11.57 ± 1.54	
	Diploma	104 (49.52)	12.58 ± 1.39	
	Bachelor	35 (11.90)	12.31 ± 1.14	
	Master’s degree and higher	13 (13.34)	12.05 ± 1.62	
Father’s job	Self-employed	102 (48.57)	12.41 ± 1.24	0.814**
	Employee	73 (34.76)	11.13 ± 1.57	
	Unemployed	35 (11.90)	12.78 ± 1.34	
Marital status	Married	198 (94.28)	10.98 ± 1.68	0.017*
	Divorce	12 (5.72)	14.58 ± 1.14	
Number of children	1	87 (41.42)	14.64 ± 1.39	0.018**
	2	104 (49.53)	11.08 ± 1.52	
	3 and more	19 (9.05)	10.04 ± 1.13	
Sex of children	Boy	121 (57.61)	12.43 ± 1.21	0.832*
	Girl	89 (42.39)	12.57 ± 1.54	
Children’s age	3–6	58 (27.62)	13.58 ± 1.39	0.858**
	7–11	73 (34.76)	13.31 ± 1.14	
	12–14	79 (37.62)	12.95 ± 1.62	
Duration of the child’s disease (Year)	1–3	73 (34.76)	10.58 ± 1.59	0.021**
	4–7	97 (46.20)	11.31 ± 1.74	
	8 and more	40 (19.04)	14.95 ± 1.24	
Kind of disease	Thalassemia	22 (10.48)	13.98 ± 1.53	0.841**
	Cancer	74 (35.24)	13.58 ± 1.27	
	Heart disease	42 (20.0)	13.44 ± 1.21	
	Trauma	72 (34.28)	13.72 ± 1.48	

*Independent *t*-test. **ANOVA test.

### 3.2. Death anxiety, resilience, and family cohesion in the parents of children and adolescents in the end stages of life

The death anxiety, resilience, and family cohesion mean scores of the parents who participated in the present study were 14.13 ± 1.25, 58.99 ± 2.32, and 71.83 ± 2.44, respectively (Table 2).

**Table 2:** The means and standard deviations of the participants’ death anxiety, family adaptability cohesion, and resilience.

Variable	Dimensions	Mean ± SD (Each dimension)	Mean ± SD (Total)
Death anxiety	Without dimension	14.13 ± 1.25	14.13 ± 1.25
Family adaptability cohesion	Cohesion	74.28 ± 2.31	71.83 ± 2.44
	Adaptability	69.39 ± 2.58	
Resilience	Individual competence	56.65 ± 2.23	58.99 ± 2.32
	Tolerating the negative effects and stress	62.87 ± 1.94	
	Acceptance of change	51.54 ± 2.05	
	Self-control	61.77 ± 2.74	
	Spiritual effects	62.15 ± 2.64	

Mean ± SD, Mean ± Standard Deviation.

### 3.3. The relationship between death anxiety, resilience, and family cohesion in the parents of children and adolescents in the end stages of life

The findings of the study showed that there was a significant inverse relationship between death anxiety on the one hand and resilience (*p* < 0.001, *r* = −0.92) and family cohesion (*p* < 0.001, *r* = −0.90) on the other. In addition, the participants’ resilience mean score was found to have a significant direct relationship with their family cohesion mean score (*p* < 0.001, *r* = 0.87; Table 3).

**Table 3:** Relationship between death anxiety, family adaptability cohesion, and resilience in parents.

Death anxiety	Resilience	*r* = −0.92	*p* < 0.001
Death anxiety	Family adaptability cohesion	*r* = −0.90	*p* < 0.001
Resilience	Family Adaptability Cohesion	*r* = 0.87	*p* < 0.001

r, Pearson test.

### 3.4. Predictive factors of death anxiety in the parents of children and adolescents in the end stages of life

The variables of resilience, family cohesion, marital status, number of children, and children’s illness duration which had *p* < 0.25 were entered into multivariate linear regression with the backward technique. Demographic variables and resilience, family cohesion have been included in the regression model according to the fact that they have the highest correlation with death anxiety. These variables remained in the model and accounted for 61.34% of the death anxiety variance in the parents of children and adolescents in the end stages of life (Table 4).

**Table 4:** The predictor variables of death anxiety in parents of children and adolescents in the end stages of life.

Factors		Non-standard coefficients		Standard coefficients	*T*	*p*-value
		B	Standard deviation	*β*		
Resilience		1.68	2.78	2.31	2.07	0.001
Family adaptability cohesion		1.64	2.57	1.86	2.11	0.001
Marital status	Married	Ref	–	–	–	–
	Divorce	1.27	1.43	1.23	1.05	0.017
Number of children	1	Ref	–	–	–	–
	2	1.29	1.23	1.58	1.08	0.018
	3 and more	1.41	1.74	1.27	1.14	0.020
Duration of the child’s disease	1–3	Ref	–	–	–	–
	4–7	1.18	2.12	1.75	1.21	0.023
	8 and more	1.27	2.42	1.23	1.76	0.021

Adjusted *R*^2^: 61.34%.

## 4. Discussion

In the present study, the parents of children and adolescents in the end stages of life were found to have high levels of death anxiety, low levels of resilience, and moderate levels of family adaptability and cohesion. There was a significant inverse correlation between death anxiety, on the one hand, and resilience and family cohesion, on the other hand, but there was a significant direct correlation between resilience and family cohesion. Although several studies have addressed work stress, knowledge, and awareness in the caregivers of adult patients in end-of-life stages (Khalvati et al., 2021; Rababa et al., 2021; Upenieks, 2021), only a few studies have explored death anxiety, resilience, and family cohesion in the parents of children and adolescents with chronic illness or after the death of their children and adolescents (Wikman et al., 2018; Koca et al., 2019; Cao et al., 2020; Dadfar et al., 2021). Therefore, the researchers had to use articles on death anxiety, resilience, and family adaptability and cohesion in other groups of patients and their caregivers or the mothers of disabled children in the end stages of life.

The death anxiety mean score of the parents of children and adolescents in the end-of-life stage was 14.13 ± 1.25, which indicates considerable death anxiety in the parents as the main caregivers of these patients. As a significant factor in caring for children and adolescents in the end stages of life, death anxiety was found to be influenced by parents’ number of children, marital status, and illness duration of their children. On a similar note, other studies reported that the caregivers, especially the parents, of end stages of life children experience high levels of death anxiety (Wikman et al., 2018; Koca et al., 2019; Dadfar et al., 2021). According to Wikman et al. (2018), the parents, especially the mothers, who lost a child to cancer experienced high levels of death anxiety and depression for 5 years after the death of their child. The parents’ age, gender, emotional health, resilience, and depression had an impact on their death anxiety. This finding is consistent with the results of the present study; however, the parents in the present study experienced much greater degrees of death anxiety than the parents in Wikman’s study. This discrepancy can be attributed to the fact that Wikman measured parents’ anxiety about the death of their children over a 5-year period; evidently, time can alleviate bereaved parents’ anxiety and psych-emotional tension (Wikman et al., 2018). As with the present study, a study by Koca et al. (2019) showed that the mothers of children with progressive disorders in bad health conditions experience considerable death anxiety. Also, the results showed that the mothers’ number of children, marital status, and financial status had an impact on their death anxiety. The death anxiety mean score of the mothers in Koca’s study was lower than in the present study; the discrepancy can be attributed to differences between the study populations and their living conditions. It is evident that the parents of children in end-of-life stages experience greater death anxiety than the parents of children with progressive disorders (Koca et al., 2019). Dadfar et al. reported that patients in end-of-life stages are exposed to high levels of death anxiety, with elderly patients experiencing greater anxiety. Since an individual’s exposure to death anxiety is influenced by his/her personal beliefs, culture, and religion, there is a need for more research in this area in various cultures (Dadfar et al., 2021).

In the present study, the resilience mean score of the parents was found to be a low 58.99 ± 2.32. The results also showed a significant inverse relationship between the death anxiety and resilience of the parents of children and adolescents in the end-of-life stage. Similarly, Cao et al. (2020) reported that bereaved mothers have great anxiety and low resilience and that there is an inverse relationship between these parents’ resilience and the anxiety caused by the death of their children: those with less resilience experience greater anxiety (Cao et al., 2020). According to Toledano-Toledano et al. (2019), the parents of children with cancer experience considerable anxiety about their children’s incurable illness and imminent death, which adversely affects their resilience. There is an inverse relationship between these parents’ resilience and death anxiety. Toledano’s study investigated anxiety and resilience in the parents of children with cancer. Even though those children were not in good physical conditions, they were not in the end-of-life stage. Accordingly, despite the fact that their parents felt great anxiety and had low resilience, they felt less anxiety and had more resilience than the parents in the present study (Toledano-Toledano et al., 2019).

The family cohesion mean score of the parents in the present study was found to be 71.83 ± 2.44 and had an inverse relationship with their death anxiety, but a direct relationship with their resilience. On a similar note, a study by Vegsund et al. (2019) showed that the death of children with cancer led to diminished resilience and cohesion in their families and that there was a direct relationship between the parents’ resilience and family cohesion after the children’s death. The family cohesion mean score of the parents in Vegsund’s study was lower than in the present study, which may be due to the fact that the former study measured family cohesion 2–8 years after the children’s death in a cultural context different from the dominant culture in Iran. Cultures, beliefs, and religious attitudes influence parents’ resilience and family cohesion (Vegsund et al., 2019). Likewise, Park et al. (2018) found that, after their children’s death, parents experience great anxiety, low resilience, and moderate family cohesion. They also found a direct relationship between resilience and family cohesion and an inverse relationship between family cohesion and death anxiety in the parents. This study was conducted in a cultural context different from the Iranian culture, which underscores the impact of the death of children on their parents and family cohesion, stressing the need for emotional support for the parents of children in the end-of-life stage (Park et al., 2018).

In finally, the findings of the present study show that the parents of end stages of life children experience severe death anxiety in the course of their children’s illness, especially in the last few months leading to their children’s death, with adverse effects on their resilience and family cohesion. Therefore, nurses caring for adolescents in the final stages of life should be aware of the psychological and physical needs of the parents of these adolescents and take action by examining the level of anxiety in order to improve their resilience and improve family cohesion.

## 5. Limitations

One of the limitations of the present study was the small sample size, so, it is suggested that future research measure death anxiety, resilience, and family cohesion in the parents of children and adolescents in end of life with a larger sample size in various societies to achieve a more accurate evaluation of death anxiety in this population. Another limitation of this study was the lack of access to the participant face-to-face and online distribution of questionnaires. Therefore, it is suggested that in future studies, in order to obtain more comprehensive information, questionnaires should be completed face-to-face and other methods of data collection should be used, including observation and focus groups.

## 6. Conclusion

The parents in the present study were suffering from great anxiety about the imminent death of their end stages of life children. The variables of resilience, family cohesion, number of children, illness duration of their children, and marital status were found to have an impact on the parents’ death anxiety: they predicted 61.34% of their death anxiety variance. So, healthcare policymakers and administrators can use these findings to develop more effective plans toward supporting these parents and improving their quality of life.

## Data availability statement

The original contributions presented in the study are included in the article/supplementary material, further inquiries can be directed to the corresponding author.

## Ethics statement

All participants signed the informed consent to participate in the study. The present study was conducted in accordance with the principles of the revised Declaration of Helsinki, a statement of ethical principles which directs the physicians and other participants in medical research involving human subjects. The participants were assured of their anonymity and confidentiality of their information Moreover, the study was approved by the local Ethics Committee of Fasa University of Medical Sciences (Ethical code: IR.FUMS.REC 1401.029). The patients/participants provided their written informed consent to participate in this study.

## Author contributions

FM and MB were responsible for data collection. FM, MB, LN, and KO drafted and provided critical revision of the manuscript. SZ and KO were responsible for designing the research protocol and data analysis. All authors read and approved the final manuscript.

## Conflict of interest

The authors declare that the research was conducted in the absence of any commercial or financial relationships that could be construed as a potential conflict of interest.

## Publisher’s note

All claims expressed in this article are solely those of the authors and do not necessarily represent those of their affiliated organizations, or those of the publisher, the editors and the reviewers. Any product that may be evaluated in this article, or claim that may be made by its manufacturer, is not guaranteed or endorsed by the publisher.

## References

[ref1] Ahmadi Farsani M. Heshmati R. Hashemi Nosrat Abad T. Rezazadeh S. (2020). The comparison of attitude toward death and anxiety sensitivity between adolescents with cancer and normal adolescents. IJCA 1, 29–37. doi: 10.29252/ijca.1.3.29

[ref2] Cafferkya J. Banbury S. Athanasiadou-Lewis C. (2018). Reflecting on parental terminal illness and death during adolescence: an interpretative phenomenological analysis. Interpersona 12, 180–196. doi: 10.5964/ijpr.v12i2.306

[ref3] Cao X. Yang C. Wang D. (2020). The impact on mental health of losing an only child and the influence of social support and resilience. Omega J. Death and Dying. 80, 666–684. doi: 10.1177/0030222818755284, PMID: 29380659

[ref4] Cong C. W. Tan C. S. Noew H. S. Wu S. L. (2022). Psychometric evaluation of the Malay version of the family adaptability and cohesion evaluation scale III for Malaysian adolescents. Int. J. Environ. Res. Public Health 19:156. doi: 10.3390/ijerph19010156 PMC8750947 35010416

[ref5] Connor K. M. Davidson J. R. (2003). Development of a new resilience scale: the Connor-Davidson resilience scale (CD-RISC). Depress. Anxiety 18, 76–82. doi: 10.1002/da.10113 12964174

[ref6] Dadfar M. Lester D. Abdel-Khalek A. M. Ron P. (2021). Death anxiety in Muslim Iranians: a comparison between youths, middle adults, and late adults. Illn. Crisis Loss 29, 143–158. doi: 10.1177/1054137318790080

[ref7] Fayand J. Akbari M. Moradi O. Karimi K. (2019). Investigating the effectiveness of resiliency pattern on improving the quality of life of multiple sclerosis patients: a follow up study. IJRN 5, 31–38. doi: 10.21859/ijrn-05035

[ref8] Garcia-Dia M. J. DiNapoli J. M. Garcia-Ona L. Jakubowski R. O'Flaherty D. (2013). Concept analysis: resilience. Arch. Psychiatr. Nurs. 27, 264–270. doi: 10.1016/j.apnu.2013.07.003 24238005

[ref9] Holakouie-Naieni K. Koehler S. A. Karimi R. Mardani F. Karimi J. (2016). Unnatural deaths among children and adolescents in Isfahan Province, Iran: a forensic epidemiology study of postmortem data. J. Forensic Nurs. 12, 90–94. doi: 10.1097/JFN.0000000000000114 27195930

[ref10] Javalkar K. Rak E. Phillips A. Haberman C. Ferris M. van Tilburg M. (2017). Predictors of caregiver burden among mothers of children with chronic conditions. Children 4:39. doi: 10.3390/children4050039, PMID: 28509853 PMC5447997

[ref11] Khalvati M. Babakhanian M. Khalvati M. Nafei A. Khalvati M. Ghafuri R. (2021). Death anxiety in the elderly in Iran: a systematic review and meta-analysis. Iran. J. Ageing 16, 151–171. doi: 10.32598/sija.16.2.862.2

[ref12] Koca A. Basgul S. S. Yay M. (2019). Comparison of death anxiety and state-trait anxiety levels in mothers of disabled children and non-disabled children. Dusunen Adam. 32, 58–64. doi: 10.14744/DAJPNS.2019.00008

[ref13] Langton C. E. Berger L. M. (2011). Family structure and adolescent physical health, behavior, and emotional well-being. Soc. Serv. Rev. 85, 323–357. doi: 10.1086/661922, PMID: 23788821 PMC3685438

[ref14] Lerch M. F. Thrane S. E. (2019). Adolescents with chronic illness and the transition to self-management: a systematic review. J. Adolesc. 72, 152–161. doi: 10.1016/j.adolescence.2019.02.010, PMID: 30903932

[ref15] Mohammadi M. R. Ahmadi N. Salmanian M. Asadian-Koohestani F. Ghanizadeh A. Alavi A.. (2016). Psychiatric disorders in Iranian children and adolescents. Iran. J. Psychiatry 11, 87–98. 27437005 PMC4947225

[ref16] Mohammadi F. Masoumi Z. Oshvandi K. Khazaei S. Bijani M. (2021). Death anxiety. Moral courage, and resilience in nursing students who care for COVID-19 patients: a cross-sectional study. BMC Nurs. 12, 1–7. doi: 10.1186/s12912-022-00931-0 PMC9189788 35698221

[ref001] Olson D. H. (2011). FACES IV and the circumplex model: validation study. J. Marital Fam. 37, 64–80. 10.1111/j.1752-0606.2009.00175.x 21198689

[ref18] Park Y. Y. Jeong Y. J. Lee J. Moon N. Bang I. Kim H.. (2018). The influence of family adaptability and cohesion on anxiety and depression of terminally ill cancer patients. Support. Care Cancer 26, 313–321. doi: 10.1007/s00520-017-3912-4, PMID: 28975413

[ref19] Phillips B. E. Theeke L. A. Sarosi K. M. (2020). Relationship between negative emotions and perceived support among parents of hospitalized, critically ill children. Int J Nurs Sci. 8, 15–21. doi: 10.1016/j.ijnss.2020.10.001, PMID: 33575440 PMC7859540

[ref20] Pinquart M. (2020). Health-related quality of life of young people with and without chronic conditions. J. Pediatr. Psychol. 45, 780–792. doi: 10.1093/jpepsy/jsaa052 32642762

[ref21] Rababa M. Hayajneh A. A. Bani-Iss W. (2021). Association of death anxiety with spiritual well-being and religious coping in older adults during the COVID-19 pandemic. J. Relig. Health 60, 50–63. doi: 10.1007/s10943-020-01129-x, PMID: 33284402 PMC7719733

[ref22] Reif L. K. van Olmen J. McNairy M. L. Ahmed S. Putta N. Bermejo R.. (2022). Models of lifelong care for children and adolescents with chronic conditions in low-income and middle-income countries: a scoping review. BMJ Glob. Health 7:e007863. doi: 10.1136/bmjgh-2021-007863 PMC9255401 35787510

[ref23] Scoloveno R. (2016). A concept analysis of the phenomenon of resilience. J. Nurs. Care 5, 1–5. doi: 10.4172/2167-1168.1000353

[ref24] Sharif Nia H. Pahlevan Sharif S. Goudarzian A. H. Haghdoost A. A. Ebadi A. Soleimani M. A. (2016). An evaluation of psychometric properties of the Templer’s death anxiety scale-extended among a sample of Iranian chemical warfare veterans. J. Hayat 22, 229–244.

[ref25] Toledano-Toledano F. Moral de la Rubia J. Broche-Pérez Y. Domínguez-Guedea M. T. Granados-García V. (2019). The measurement scale of resilience among family caregivers of children with cancer: a psychometric evaluation. BMC Public Health 19, 1–4. doi: 10.1186/s12889-019-7512-8 31455340 PMC6712960

[ref26] Tomás-Sábado J. Gómez-Benito J. (2002). Psychometric properties of the Spanish form of templer’s death anxiety scale. Psychol. Rep. 91, 116–120. doi: 10.2466/pr0.2002.91.3f.1116 12585522

[ref27] Upenieks L. (2021). Uncertainty in faith, fear of death? Transitions in religious doubt and death anxiety in later life. Omega J. Death and Dying 5:00302228211029475 doi: 10.1177/00302228211029475 34225499

[ref28] Vegsund H. K. Reinfjell T. Moksnes U. K. Wallin A. E. Hjemdal O. Eilertsen M. E. (2019). Resilience as a predictive factor towards a healthy adjustment to grief after the loss of a child to cancer. PLoS One 14:e0214138. doi: 10.1371/journal.pone.0214138, PMID: 30897157 PMC6428287

[ref29] Wijlaars L. P. M. M. Gilbert R. Hardelid P. (2016). Chronic conditions in children and young people: learning from administrative data. Arch. Dis. Child. 101, 881–885. doi: 10.1136/archdischild-2016-310716, PMID: 27246068 PMC5050282

[ref30] Wikman A. Mattsson E. von Essen L. Hovén E. (2018). Prevalence and predictors of symptoms of anxiety and depression, and comorbid symptoms of distress in parents of childhood cancer survivors and bereaved parents five years after end of treatment or a child's death. Acta Oncol. 57, 950–957. doi: 10.1080/0284186X.2018.1445286 29498559

[ref31] Windle G. (2011). What is resilience? A review and concept analysis. Rev. Clin. Gerontol. 21, 152–169. doi: 10.1017/S0959259810000420

